# Prof. Richard T Silver—Pioneer of MPN 18 January, 1929–17 April, 2026

**DOI:** 10.1038/s41375-026-03008-y

**Published:** 2026-06-26

**Authors:** Rüdiger Hehlmann

**Affiliations:** Medizinische Fakultät Mannheim, Universität Heidelberg, ELN Foundation Weinheim, Mannheim, Germany

**Keywords:** Myeloproliferative disease, Leukaemia

Prof Richard Silver from Weill-Cornell in New York (Dick for his friends) is known for his work on myeloproliferative neoplasms (MPN). He started exploring a possible benefit of recombinant interferon α on polycythemia vera (PV) in the late 1980ties [1], when treatment consisted of phlebotomy and hydroxyurea, and on chronic myeloid leukemia (CML) after developing an evidence-based definition of CML-blast-crisis by 30% blasts in the blood, which is still in use [2]. His research was supported by the Cancer Research & Treatment Fund, which he farsightedly had founded in 1968 for donations by grateful patients. He became internationally visible when he chaired an expert panel of the American Society of Hematology (ASH) to prepare an evidence-based analysis for treating CML (1999) [3]. When the professional writer gave notice because of budget reasons, and ASH was about to revoke the project, Dick, with the determination and persistance characteristic of his personality, decided to do the writing himself. In 2001, he started, together with Jerry Spivak from Johns Hopkins, Baltimore, a series of international conferences, initially on MPN and CML, later on MPN, which became highly successful. He convened 16 conferences, the last one in 2024. The first conference in October 2001 was convened only a month after the 9/11 events. Despite many cancellations, the conference took place as expected by those who knew Richard’s personality. The conference became a central opportunity to discuss internationally the tremendous progress made in the MPNs following the detection of molecular markers as therapeutic targets and for monitoring treatment.

**Figure:**
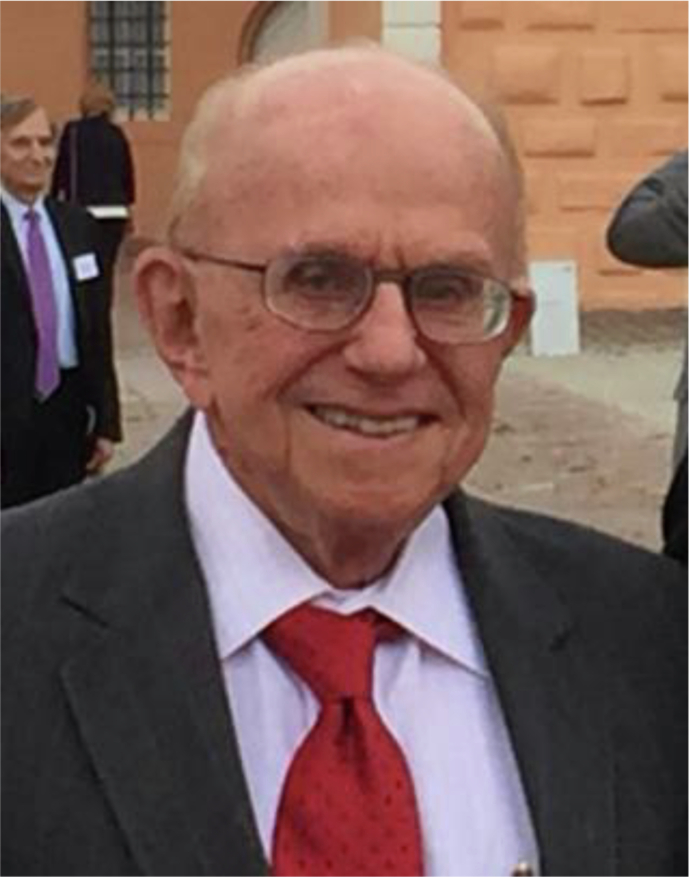

The conference was accompanied by an educational meeting specifically for patients as a tribute to his many grateful patients.

As a clinical scientist and knowledgeable, cooperative colleague and friend, he was invited to join the panel of the European LeukemiaNet (ELN) for the preparation of ELN-management recommendations for treating CML [4]. He contributed to 5 editions, the last one in 2025. He succeeded to bring his institution into the ELN, thus Cornell University becoming the first US participant of ELN, to be followed by 3 others (Ohio State, Emory, Ann Arbor). In 2011 his achievements were honoured by Weill-Cornell with the establishment of an RT Silver-Center on Myeloproliferative Neoplasms. The Center serves as a cristallization point for Dick’s junior colleagues (Joseph Scandura, Gaith Abu-Zeinah, others) working on MPN. In 2019, Richard received the ELN-Merit Award for international integration of leukemia (MPN) research.

There are some remarkable features of Richard’s career: he was born and educated in New York, he worked there all his life in his private practice at York/77th Street up to age 70 and at Weill-Cornell, also after retirement at age 90, and he died in New York. He was a New Yorker! He left New York only for medical service in the Korean War and as a visiting Fulbright professor to establish a residency program at Bahia University in Salvador, Brazil.

During that time, he visited the indigenous Indian population at the upper Xingu River, a tributory of the Amazon, and detected a new rare blood subgroup in this population [5]. As a consequence, he was elected to the renowned Explorer’s Club in New York. At the occasion of an early MPN conference, Dick invited the faculty to the Explorer’s Club at Manhattan’s Upper Eastside to meet an astronaut, also a member of the Explorer’s Club, in person. I still remember this spectacular event, although the European faculty, due to the time difference, could not fully appreciate the astronaut’s report.

Another remarkable feature is that he started the career he is internationally known for rather late in life. Dick was in his late 60s when he chaired the ASH panel on CML, and he was 72 when he convened his first MPN conference. He rarely mentioned his age, and thus, due to his vitality, could easily go for somebody 10 years younger. He regularly attended the ASH conferences, last in 2024 in San Diego, including the President’s receptions, the MPN-Heros‘ Dinners and the ELN-Breakfast meetings Sunday mornings at 6 AM. His 95th birthday, in January 2024, was celebrated at his university with a formal dinner and an academic event with international faculty. Although his physical and intellectual fitness were remarkable, his eyesight deteriorated and became his major limitation. In 2025, at age 96, he handed the MPN conference over to Jerry Spivak and 2 younger colleagues, John Mascarenhas from Mt. Sinai, N.Y., and Naveen Pemmaraju from MDACC, Houston, with Heather Newton from Melton Medical Education as the organizer. I saw him last in November 2025. He had lost weight, but intellectually he was fit as ever.

Richard Silver’s greatest achievements are the early recognition of the beneficial effect of interferon α in PV and the establishment of an academic MPN-center in connection with an international MPN-conference.

Dick is survived by his beloved wife Barbara of more than 60 years, his son Adam and his granddaughters Stella and Isla.

Richard is remembered by the MPN-community as a pioneer of MPN, by me as a great friend and mentor.
